# Quercetin Alleviates Red Bull Energy Drink-Induced Cerebral Cortex Neurotoxicity via Modulation of Nrf2 and HO-1

**DOI:** 10.1155/2021/9482529

**Published:** 2021-10-31

**Authors:** Walaa Mohamed Sayed

**Affiliations:** Department of Anatomy and Embryology, Faculty of Medicine, Kasr Al-Ainy, Cairo University, Cairo, Egypt

## Abstract

The current work was aimed at evaluating the ameliorative role of quercetin (QR) on the possible toxicity of Red Bull energy drink (RB-Ed) in the cerebral cortex of rats. To achieve the goal, the rats were allocated into 4 groups. The first group received distilled water as control. Groups II and III were given Red Bull energy drink alone and in combination with quercetin, respectively. The fourth group served as recovery to group II. The experimental duration was four weeks for all groups whereas the recovery period of group IV was two weeks. QR upregulated the mRNA and protein expression levels of nuclear factor erythroid 2-related factor 2 (Nrf2) and heme oxygenase-1 (HO-1) genes, which can protect against RB-Ed neurotoxicity. Moreover, by reducing the thiobarbituric acid reactive substance and increasing the total antioxidant capacity levels, QR protected rats' cerebral cortex against Red Bull energy drink-induced oxidative damage. Quercetin also inhibited RB-Ed-induced histomorphological degeneration which was confirmed by the increase in the intact neurons and the rise in the neuron-specific enolase immunoreaction. QR increased the reduction of the brain-derived neurotrophic factor that was elicited by RB-Ed and acts as an anti-inflammatory agent by reducing the proinflammatory marker, interleukin 1 beta and DNA damage markers, heat shock protein 70, and 8-hydroxydeoxyguanosine. It could be concluded that the alleviating impacts of QR on RB-Ed neurotoxicity in rats could be related to the modulation of Nrf2 and HO-1 which in turn affects the redox status.

## 1. Introduction

Energy drinks (Eds) are carbonated beverages that are popular among people of all ages all over the world. Individuals believe that consuming Eds can improve their performance by providing them with more energy, so they consume a lot of Eds every day [1]. Several companies compete to manufacture energy drinks and market these Eds as activators with appealing names that convey strength, power, and speed; the most popular brand names in Egypt are Red Bull and Power Horse [2].

The stimulating ingredients in the Red Bull energy drink (RB-Ed) include caffeine, vitamin B complex, panthenol, carbohydrates, simple sugars, niacin, glucuronolactone, and taurine amino acids [3], as well as taste-like suppliers such as ginseng, guarana, ephedrine, and ginkgo [4]. The active components in most Eds have a significant effect on human body metabolism and mental energy construction, which improves activity and concentration while studying and driving, overcomes sleepiness, and reduces headache symptoms [1, 5].

Following the death of Ross Coony, a sportsperson, due to his consumption of four cans of RB-ED before the start of play, France banned the popular Eds, particularly Red Bull (RB), and Brittan issued a warning about RB-Ed consumption by pregnant women and children [6]. Eds have several negative side effects, including cardiac arrhythmia, hypertension [7], irritability, lethargy, mental misperception, concentration problems, and intellectual functioning issues as well as hepatic, renal, and cardiovascular adverse effects [1, 5], in addition to neurologic complications [8]. The mechanisms by which RB-Ed induce structural injuries of the rat cerebral cortex is not completely comprehended. Oxidative stress has been linked to the mechanism of RB-Ed-induced injurious effects, so the utilization of antioxidants may help [9].

Lately, the protective characteristics of flavonoids such as quercetin (QR) have obtained growing attention. QR is a natural flavonoid found in a variety of fruits and vegetables, the highest concentration being in onions [10]. QR acts as an antioxidant to protect against cardiovascular, infectious, gastrointestinal, and renal diseases [11] as well as neuronal degeneration diseases [12] via stimulation of the nuclear factor erythroid 2-related factor 2 (Nrf2). The latter is involved in the antioxidative and anti-inflammatory mechanisms [13] by stimulation of heme oxygenase-1 (HO-1), an antioxidant enzyme [14].

RB-Ed causes structural changes in the rat cerebral cortex, such as neuronal and nuclear atrophy [15]. Because neuron-specific enolase (NSE) is abundant in neurons of both the central nervous system (CNS) and the neuroendocrine system [16], it is used as a marker for cerebral neurodegeneration in a wide range of CNS disorders [17]. Additionally, according to Abdel-Wahab and Metwally [18], the immunohistochemical anti-NSE antibody stain is only found in the CNS. Furthermore, brain-derived neurotrophic factor (BDNF) is predominantly found in neuronal cells, allowing them to adapt and respond to changes in architecture and action [19].

No consensus has been reached on the adverse effects of Red Bull energy drink on the cerebral cortex, and studies investigating the mechanism of action of quercetin in the amelioration of RB-induced cerebral cortex neurotoxicity are lacking. This study is aimed at investigating the beneficial properties of quercetin as well as its potential role in modulating Nrf2 and HO-1 to alleviate RB-Ed-induced oxidative stress and neurotoxicity of the cerebral cortex architecture.

## 2. Material and Methods

### 2.1. Animals

The sample size was calculated using G∗power 3.1.9.4 and twenty Wistar adult male albino rats weighing between 190 and 240 g were utilized in the experiment. All rats were kept in metal cages at Cairo University's Faculty of Medicine's Animal and Experimental House, where they had free access to water and food in a controlled environment (22 ± 2°C) with a 12-hour/12-hour light/dark cycle. The rats were given a 5-day acclimatization period before the experiment began. The experiment was authorized by Cairo University's Institutional Animal Care and Use Committee and carried out in accordance with the National Institutes of Health's Guide for the Care and Use of Laboratory Animals. To minimize the animals' suffering, all animal procedures and treatments were properly managed.

### 2.2. Chemicals

Red Bull energy drink (RB-Ed) in the form of a can (250 mL) was obtained from a local market in Cairo, Egypt. RB-Ed was administered at a dose of 10 mg/kg/day (7.5 mL) [20]. Each 100 mL of RB-Ed contains simple sugars (sucrose and glucose), water, citric acid, sodium citrate, carbon dioxide, inositol (0.02%), taurine amino acid (0.4%), caffeine (0.03%), pantothenic acid (2 mg), niacin (8 mg), B12 (0.002 mg), vitamin B6 (2 mg), riboflavin, flavors, caramel, and tinting elements. Quercetin (QR) was purchased from Sigma-Aldrich (St Louis, MO, USA). The daily dose of quercetin was 50 mg/kg/day [21].

### 2.3. The Experimental Protocol

The rats were assigned into 4 groups (*n* = 5, each). Group I (control group) received distilled water. Groups II and III were given Red Bull energy drink alone and in combination with quercetin, respectively. Group IV served as recovery to group II. All treatments were administered daily via oral gavage. The experimental duration was four weeks for all groups while the recovery period of group IV was two weeks.

After euthanization, the brains were carefully removed and cleaned in a cold normal saline solution (0.9%). On an ice glass plate, each brain was carefully separated into two cerebral hemispheres. Fresh samples from the cerebral cortex were collected and washed with refrigerated saline. Homogenization was performed in ice-cold phosphate-buffered saline (PBS, 100 mM, pH 7.4) by using a cut-glass tissue Teflon homogenizer (Ultra Turrax IKA T18 Basic). All homogenates were then centrifuged at 4000 rpm for 15 min at 4°C. The supernatant was separated, frozen immediately, and then kept at −80°C until the time of biochemical and molecular analyses. Samples of the brain tissue were preserved in 10% neutral-buffered formalin solution overnight, dehydrated in increasing grades of ethanol, cleared in xylene, and embedded in paraffin wax for histopathological and immunohistochemical investigations [22].

### 2.4. Measurement of the Redox Status in the Cerebral Cortex

By using a thiobarbituric acid reactive substance (TBARS) assay ELISA kit, the lipid peroxidation state of cerebral cortex homogenates was determined (Zymed, Carlton Court, San Francisco). The concentration of TBARS in cerebral cortex tissue protein was represented in micromoles per milligram (*μ*M/mg). The total antioxidant capacity (TAC) of cerebral cortex homogenates was tested and represented in micromoles per milligram of cerebral tissue protein (*μ*M/mg) using the total antioxidant capacity kit (Cell Biolabs, Inc., San Diego, CA, USA) [23].

### 2.5. Real-Time Polymerase Chain Reaction Analysis of mRNA Expression of Nuclear Factor Erythroid 2-Related Factor 2 (Nrf2) and Heme Oxygenase-1 (HO-1) Genes

Using the Invitrogen TRIzol kit, total RNA was extracted from cerebral cortical tissues according to the manufacturer's recommendations (Thermo Fisher Scientific, USA). To reverse transcribe into cDNA, the Reverse Transcription Kit (Applied Biosystems, USA) was used. Gene-specific primer pairs were as follows: Nrf2: 5′ GTGCTATGGAGCCTTGACAT 3′ (fwd), 5′ TAATGCTCGATCTCGAGTCT 3′ (rev) and HO-1: 5′ CAGTTAACGATCGACTCGCTTC 3′ (fwd), 5′ TGCTGGTCTAAGTGCTGGACAGTCA 3′ (rev). For amplification, SYBR® Premix Ex Taq™ II Universal PCR Master Mix was utilized on an ABI 7300 Detection System (Applied Biosystems, CA, USA) (Takara, Japan). All findings were standardized to beta-actin: 5′ GTCATGCGCATAGCCTAG 3′ (fwd), 5′ CTGGTAGACCGAAGTGCTTGTG 3′ (rev). The relative expression of the genes under investigation was estimated using the comparative threshold cycle approach.

### 2.6. Western Blot Estimation of the Protein Expression Levels of Nrf2 and HO-1

An ice-cold radioimmunoprecipitation assay buffer was utilized to extract proteins from the homogenized cerebral cortex. The Bradford assay was used to assess the protein concentration in the supernatants of each sample [24]. Each sample comprised 20–30 g of total protein, which was put onto a sodium dodecyl sulphate polyacrylamide gel and separated by electrophoresis. The isolated proteins were transferred on ice to polyvinylidene difluoride membranes using a Bio-Rad Trans-Blot technology (Pierce, Rockford, IL, USA). Membranes were rinsed in PBS before being blocked for 1 h at room temperature in Tris-buffered saline (TBS) containing 5% nonfat milk. The blots were produced by incubating them with solutions of primary monoclonal mouse antibodies overnight at 4°C and pH 7.6 with gentle shaking for Nrf2 (Cat. no. MAB3925, IgG2B Clone, R&D Systems Biotechnology, USA) and HO-1 (Cat. no. Ab82585, Santa Cruz Biotechnology, USA). After that, the membranes were washed twice with TBS and incubated with the secondary antibody mix for 1 h at 37°C. After washing with TBS, membrane chemiluminescence signals were produced using a Western Bright ECL HRP (chemiluminescent) substrate (Advansta Inc., San Jose, CA, USA). Membrane chemiluminescence was imaged using the ChemiDoc MP imaging system, which was provided by Lab™ image analysis software version 5.1. (Bio-Rad Laboratories Inc., Hercules, CA, USA). The imaging system compared the band intensities of the detected proteins to beta-actin (*β*-actin) protein expression, which was employed as a loading control to estimate their amounts.

### 2.7. Determination of Brain-Derived Neurotrophic Factor in the Cerebral Cortex

To calculate the results, a rat ELISA kit for brain-derived neurotrophic factor (BDNF) (RayBiotech, Inc., USA) was used to measure BDNF concentrations in homogenates of cerebral cortex from all groups in picograms per milligrams of cerebral tissue protein (pg/mg).

### 2.8. Estimation of Interleukin 1 Beta in the Cerebral Cortex

The proinflammatory cytokine interleukin 1 beta (IL-1*β*) was measured in the rat cerebral cortex using ELISA kits (Thermo Scientific, Waltham, MA, USA) according to the manufacturer's instructions. The results were calculated using picograms per milligram of cerebral tissue protein (pg/mg).

### 2.9. Estimation of DNA Fragmentation

Heat shock protein 70 (HSP70) and 8-hydroxydeoxyguanosine (8-OHdG) markers were assessed in brain homogenates as recommended by the manufacturer's instructions using ELISA kits (Thermo Scientific, Waltham, MA, USA).

### 2.10. Micromorphological Evaluation of the Cerebral Cortex

Sections of 5-micron thickness were cut from paraffin blocks, then mounted on slides, and stained with hematoxylin and eosin before being studied under a light microscope [25]. Other slices of the cerebral cortex from all experimental groups were also stained with toluidine blue dye [26].

### 2.11. Neuron-Specific Enolase (NSE) Immunohistochemistry in Cerebral Cortical Tissues

The paraffin blocks were dewaxed and rehydrated to show the cerebral cortex tissues and then treated with hydrogen peroxide (H_2_O_2_, 3%) for 5 min before being rinsed with PBS for 15 min. to inhibit endogenic peroxidase activity. The sections were washed in PBS containing normal goat serum (NGS) (1.5%) and Triton X- (Tx-) 100 to remove the unfocused tie (0.5%). After that, the sections were treated with anti-NSE primary antibodies at room temperature (Zymed, Carlton Court, San Francisco). Biotinylated anti-IgG secondary antibodies and the streptavidin-peroxidase conjugate were added for 5 min at room temperature after rinsing in PBS-Tx, followed by H_2_O_2_ (0.6%) and diaminobenzidine (0.02%). An anti-NSE rabbit polyclonal antibody (Cat. no. AB951; dilution 1 : 250) was utilized to detect the metabolically active neurons. Finally, slides stained exclusively with the secondary antibody IgG served as negative controls [27].

### 2.12. Quantitative Histomorphometric Assessment

The number of preserved neurons in toluidine blue-stained sections [26] and the number of positive immunoreactive NSE [27] were counted in ten noncoinciding microscopic power fields of cerebral cortex tissue. Two independent observers who were uninformed of the study technique or groups inspected each segment, and a third examiner agreed and determined the number of neurons. The count was carried out using a computer-assisted image analysis system, the Leica Qwin 500 (Cambridge, UK), with the camera sensitivity (×40) and objective resolving power set to the maximum (0.65). The percentage of neuronal cells that are intact and have a positive NSE immunoreaction was calculated relative to the total number of neurons.

### 2.13. Statistical Analysis

The numerical data were reported as mean ± standard deviation (mean ± SD), and the data were analyzed using the Statistical Package for the Social Sciences (SPSS) software version 21. The one-way analysis of variance (ANOVA) was performed to compare the different groups, followed by the post hoc Bonferroni test. The statistical significance was acknowledged when the *P* value was <0.05. The percentage increase or reduction in the tested parameters was used to calculate the significant difference. The Spearman correlation coefficient was employed to determine the relationships between the variables under consideration.

## 3. Results

### 3.1. Quercetin Attenuates RB-Ed-Induced Oxidative Stress and Enhances the Antioxidant Activity in the Cerebral Cortex

**Figure 1:**
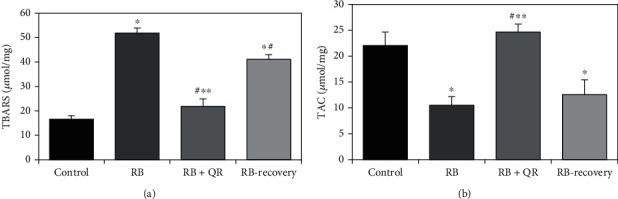
Quercetin reduced the level of TBARS (a) and increased the level of TAC (b) in the cerebral cortex of rat. ^∗^*P* < 0.01 vs. the control group; ^#^*P* < 0.01 vs. the RB group; ^∗∗^*P* < 0.01 vs. the RB-recovery group.

The TBARS level as an indicator of oxidative stress that is produced by lipid peroxidation in cerebral cortical tissues of the different experimental groups increased significantly (*P* < 0.01) in RB-treated rats when compared to either the control group or the RB+QR-cotreated group by two folds and one fold, respectively. Moreover, the statistically nonsignificant difference (*P* > 0.05) between groups III and I revealed a significant reduction in TBARS levels to normal when quercetin was given concurrently with RB. Furthermore, when the level of TBARS in group IV was compared to either normal rats or animals treated with QR in group III, there was a statistically significant increase (*P* < 0.01) by 1.5 folds and 88%, respectively. When comparing group IV to group II, TBARS concentrations decreased by 20%, which was statistically significant (*P* < 0.01), but not to the normal level (Figure 1(a)).

Administration of quercetin concurrently with Red Bull energy drink in group III resulted in an increase in the concentration of the antioxidant marker; total antioxidant capacity (TAC) in the cerebral cortex to reach the normal level as appeared from the nonsignificant difference (*P* > 0.05) when comparing the QR-cotreated group with the control one. Furthermore, the RB administration in either group II or recovery group IV resulted in a significant reduction (*P* < 0.01) in the TAC concentration as compared with either control group by 52% and 43%, respectively, or QR-administered group by 57% and 49%, respectively. On the other hand, there was a statistically nonsignificant difference (*P* > 0.05) when comparing both groups II and IV with each other (Figure 1(b)).

### 3.2. Quercetin Enhances the mRNA Expression Levels of Nrf2 and HO-1 in the Cerebral Cortex

**Figure 2:**
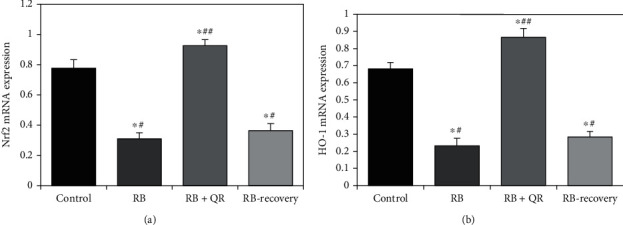
Impact of quercetin on mRNA expression level of Nrf2 (a) and HO-1 (b). ^∗^*P* < 0.01 vs. the control group; ^#^*P* < 0.01 vs. the RB+QR-cotreated group; ^##^*P* < 0.01 vs. the RB group and the RB-recovery group.

Administration of quercetin with RB in group III caused a significant upregulation (*P* < 0.01) in the mRNA expression of the Nrf2 gene in the cerebral cortex when compared to the other groups: the control group by 19%, the RB-treated group by 2 folds of increase, and the RB-recovery group by 1.5 folds of increase. When compared with the control group, Red Bull treatment significantly decreased (*P* < 0.01) the mRNA expression level of the Nrf2 gene in both groups II and IV by 60% and 53%, respectively. However, no significant difference (*P* > 0.05) existed between groups II and IV (Figure 2(a)).

When compared to either the control group or the RB+QR-cotreated group, Red Bull administration resulted in a statistically significant drop (*P* < 0.01) in the mRNA expression level of HO-1 in group II by 66% and 74%, respectively. When compared to the control rats, group III which was cotreated with QR showed a substantial upregulation (*P* < 0.01) by 27%. Furthermore, group IV, which received Red Bull treatment for 4 weeks and then rested for 2 weeks, revealed a statistically significant reduction (*P* < 0.01) in HO-1 mRNA expression when compared to the control group by 58% and the QR concomitant administration group by 67%, but no statistically significant difference (*P* > 0.05) was found when comparing group IV to group II (Figure 2(b)).

### 3.3. Quercetin Upregulates the Expression Levels of Nrf2 and HO-1 Proteins in the Cerebral Cortex

**Figure 3:**
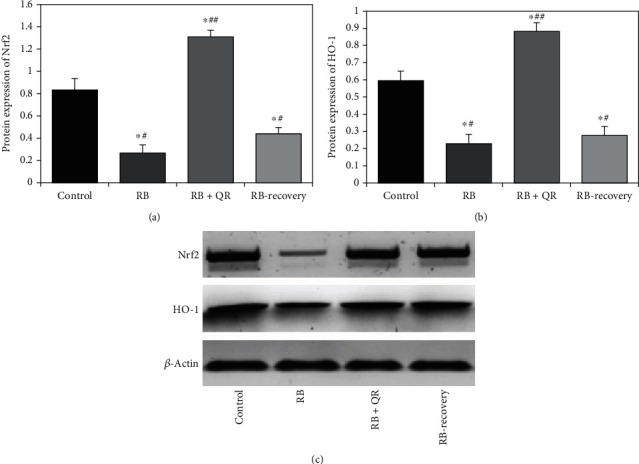
Quercetin enhances the protein expression levels of Nrf2 (a) and HO-1 (b). ^∗^*P* < 0.01 vs. the control group; ^#^*P* < 0.01 vs. the RB+QR cotreated group; ^##^*P* < 0.01 vs. the RB group and the RB-recovery group. (c) Western blot analysis of the investigated Nrf2 and HO-1 protein expression in the cerebral cortex relative to *β*-actin was imaged using the ChemiDoc MP imaging system and Lab™ image analysis software.

Concomitant administration of quercetin with RB in group III resulted in a considerable upregulation (*P* < 0.01) in the expression level of the Nrf2 protein in the cerebral cortex when compared to the other groups: control by 58%, RB-treated group by 4 folds of increase, and RB-recovery group by 2 folds of increase. When compared with the control group, Red Bull treatment substantially decreased (*P* < 0.01) the expression level of the Nrf2 protein in both groups II and IV by 69% and 47%, respectively. However, no significant difference (*P* > 0.05) existed between groups II and IV (Figures 3(a) and 3(c)).

When compared to either the control group or the RB+QR-cotreated group, Red Bull administration in group II resulted in a statistically substantial drop (*P* < 0.01) in the protein expression level of the HO-1 gene by 63% and 75%, respectively. When compared to the control rats, group III which was cotreated with QR showed a considerable upregulation (*P* < 0.01) by 49%. Furthermore, group IV, which received Red Bull treatment for 4 weeks and then rested for 2 weeks, revealed a statistically significant reduction (*P* < 0.01) in the expression level of the HO-1 protein when compared to the control group by 54% and the QR concomitant administration group by 69%, but no statistically significant difference (*P*>0.05) was found when comparing the recovery group to group II (Figures 3(b) and 3(c)).

### 3.4. The Proinflammatory Effect of RB-Ed on the Cerebral Cortex and Counteraction by Quercetin

**Figure 4:**
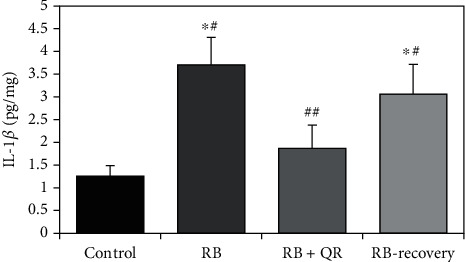
Quercetin counteracts the proinflammatory effect of RB-ED on IL-1*β* in the cerebral cortex. ^∗^*P* < 0.01 vs. the control group; ^#^*P* < 0.01 vs. the RB+QR-cotreated group; ^##^*P* < 0.01 vs. the RB group and the RB-recovery group.

To explore the potential anti-inflammatory impact of the quercetin-mediated neuroprotection in Red Bull-intoxicated rats, the level of the proinflammatory cytokine interleukin 1 beta (IL-1*β*) was measured in the cerebral cortices. Although RB administration caused significant construction (*P* < 0.01) of IL-1*β* in the cerebral cortex of rats of group II treated with Red Bull and group IV left for recovery after daily Red Bull administration for 4 weeks relative to the control group, by 2 folds and 1.4 folds of increase, respectively, quercetin significantly downregulated (*P* < 0.01) the level of IL-1*β* when compared with either group II or IV by 50% and 37%, correspondingly. On the other hand, statistically nonsignificant differences (*P* > 0.05) were noticed when comparing either the RB+QR-cotreated group with the control one or when comparing group II with group IV (Figure 4).

### 3.5. Impact of QR and RB-Ed on the Levels of Brain-Derived Neurotrophic Factor in the Cerebral Cortex

**Figure 5:**
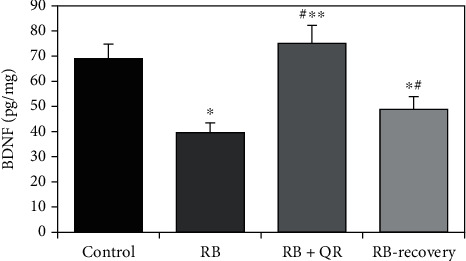
Effect of QR and RB-Ed on the level of BDNF in the cerebral cortex. ^∗^*P* < 0.01 vs. the control group; ^#^*P* < 0.01 vs. the RB group; ^∗∗^*P* < 0.05 vs. the RB-recovery group.

Red Bull energy drink intake, in either group II or recovery group IV resulted in a significant decrease (*P* < 0.01) in BDNF concentration in the cerebral cortical tissue when compared to either the control group, by 42% and 29%, respectively, or the RB+QR-cotreated group, by 47% and 35%, correspondingly. Simultaneously, in group III, concurrent treatment with quercetin resulted in a significant upregulation (*P* < 0.01) in BDNF when compared to Red Bull-treated groups II and IV, by 89% and 54%, respectively. When comparing group III to the control group, there was no significant difference (*P* > 0.05), but when comparing the RB-recovery group to the RB-treated group, there was a significant increase (*P* < 0.05) by 23% (Figure 5).

### 3.6. DNA Degradation by RB-Ed and the Moderation Role of QR

**Figure 6:**
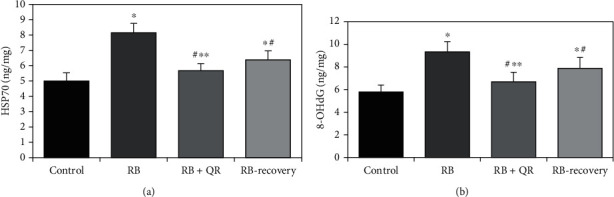
The concentrations of HSP70 (a) and 8-OHdG (b) in the cerebral cortex of all groups. ^∗^*P* < 0.01 vs. the control group; ^#^*P* < 0.01 vs. the RB group; ^∗∗^*P* < 0.05 vs. the RB-recovery group.

HSP70 concentration in the cerebral cortical tissue as a marker for DNA damage increased significantly (*P* < 0.01) in group II when compared with the normal one by 63%, the RB-recovery group by 28%, and the RB+QR-cotreated group by 44%. Administration of quercetin concomitantly with the Red Bull in group III visibly moderated the rise in the level of HSP70 relative to Red Bull-treated rats, either group II by 30% or the recovery group by 11%. Additionally, there was no significant difference (*P* > 0.05) when comparing group III with the normal one, while the RB-recovery group revealed a statistically significant reduction (*P* < 0.01) in HSP70 levels when compared with group II by 22% and a statistically significant increase when compared with the control group (*P* <0.01) by 27% and the QR-cotreated group (*P* <0.05) by 12% (Figure 6(a)).

Considering the level of 8-OHdG in the cerebral cortex of all groups, the Red Bull-treated group II revealed a statistically significant increase (*P* <0.01) in comparison with both the control group and the quercetin-cotreated group by 61% and 40%, respectively. Furthermore, the RB-recovery group showed a considerable rise in the 8-OHdG level as compared with group I (*P* < 0.01) by 36% and group III (*P* < 0.05) by 18%. On the other hand, the recovery group revealed a statistically significant decrease (*P* < 0.01) as compared with group II by 16%. The QR-cotreated group displayed a nonsignificant difference (*P* > 0.05) in comparison with the control one (Figure 6(b)).

### 3.7. Quercetin Alleviates RB-Ed Histopathological Neurodegeneration

**Figure 7:**
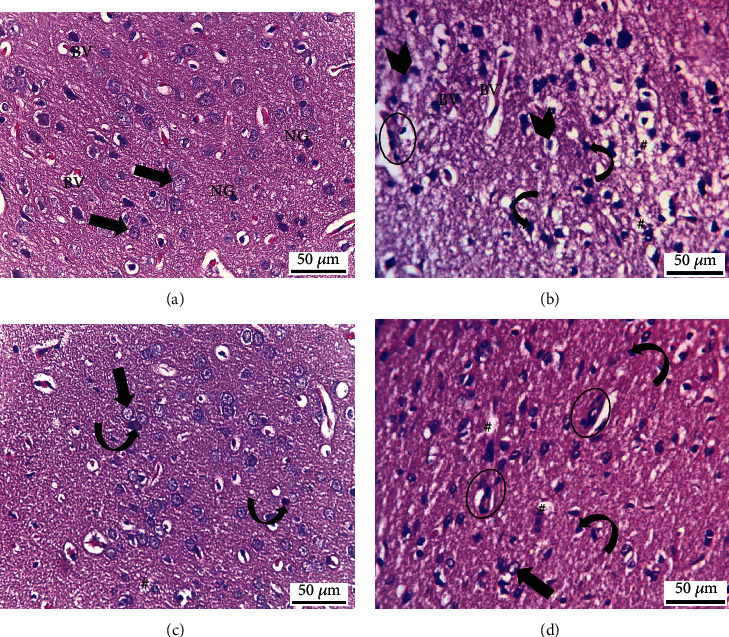
Hematoxylin and eosin-stained sections from the cerebral cortex of rats from different groups (×400, scale bars: *50 μ*m). (a) Control group displays normal architecture, cortical neurons with well-circumscribed euchromatic nuclei (arrows), and prominent nucleoli. Between the neuronal cells are neuropils, which reveal smaller nuclei of neuroglial cells (NG) and blood vessels (BV) with narrow perivascular spaces. (b) Neurons from the RB-treated group reveal degenerative histomorphological alterations with vacuoles (#) in the cytoplasm and pyknosis (curved arrows) or karyolysis (arrowheads) in the nuclei. The blood vessels (BV) appear congested, with perivascular spaces widening. There is also evidence of inflammatory cellular infiltration (circle). (c) In group III, concomitant administration of QR with the RB demonstrates an improvement of the cerebral cortex's histomorphological structure, aside from the appearance of a few cytoplasmic rarefactions (#) and scattered nuclei with either chromatin margination (arrow) or condensed chromatin alteration (curved arrows). (d) Cytoplasmic vacuoles (#) and either pyknosis (curved arrows) or marginated chromatin (arrow) of the neuronal nuclei as well as inflammatory cellular infiltration (circles) appear in the RB-recovery group.

In addition to intact neurons with euchromatic nuclei that gave normal morphological architecture, the light microscopic examination of Hx and E-stained sections of cerebral cortex from normal rats revealed blood vessels and nuclei of neuroglial cells that appeared smaller than the nuclei of the neurons in the neuropil (Figure 7(a)). On the other hand, the cerebral cortex of the Red Bull-treated group showed several neurodegenerative changes such as intracytoplasmic vacuoles of neuronal cells, darkly stained shrunken nuclei, and chromatin lysis alterations. Within the neuropil, there was also blood vessel congestion, widening of the perivascular spaces, and inflammatory changes (Figure 7(b)). Nonetheless, in group III, concomitant quercetin administration with the Red Bull significantly alleviated the RB-induced neurodegenerative alterations, as evidenced by the presence of the more established architecture of the cerebral cortex of rats with the appearance of slight degeneration, scattered areas of cytoplasmic rarefaction, and a few nuclei with either marginated chromatin or deeply stained alteration (Figure 7(c)). Moreover, there was no further improvement in the recovery group (group IV), which showed histomorphological disorganization in the cerebral cortex of rats with the appearance of both marginated chromatin and shrunken hyperchromatic in most of the nuclei of the neurons, in addition to the accumulation of inflammatory cellular infiltration (Figure 7(d)).

### 3.8. Evaluation of Intact Neurons

**Figure 8:**
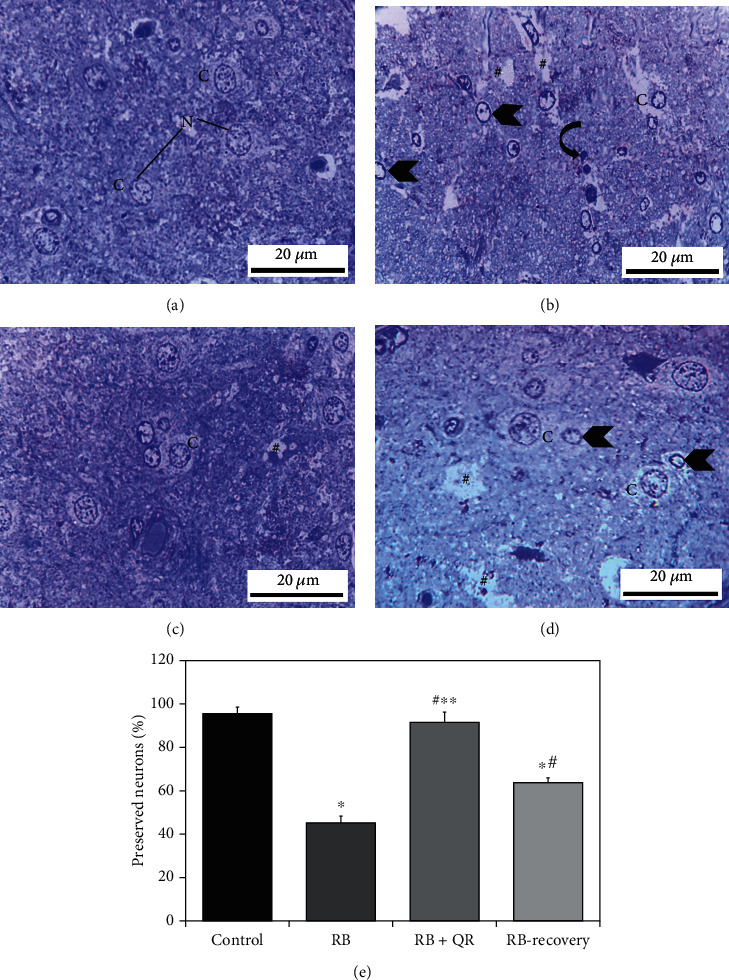
Toluidine blue-stained sections from the rat cerebral cortex of different groups (×1000, scale bars: 20 *μ*m). (a) An intact neuron with normal granular blue cytoplasm (c) and well-defined euchromatic nuclei (N) including prominent nucleoli make up the control group. (b) Group II, which had been given RB, showing large areas of degeneration (#) within the neuropil as well as a very faint cytoplasmic appearance (C). The nuclei reveal chromatin margination (arrowheads) or chromatin condensation (curved arrows). (c) In group III, QR+RB cotreatment resulted in more preserved cerebral cortex architecture, except for a few dizzy cytoplasmic neurons (C) and scattered vacuoles (#). (d) Neuropil tissue loss (#), pale neuronal cytoplasm (C), and nuclei with marginated chromatin (arrowheads) appeared in the RB-recovery group. **(**e) The percentage of preserved neurons in toluidine blue-stained sections. ^∗^*P* < 0.01 vs. the control group; ^#^*P* < 0.01 vs. the RB group; ^∗∗^*P* < 0.01 vs. the RB-recovery group.

Normal neuronal cytoplasm filled with blue granules was found in the toluidine blue-stained sections of the control group, in addition to well-established cerebral cortex architecture. Furthermore, the neuronal nuclei were encased in a distinct nuclear envelope and contained visible nucleoli as well as chromatin that was dispersed (Figure 8(a)). The cerebral cortex of the RB-treated rats in group II, on the other hand, showed extensive neuropil degeneration, as well as severely pale cytoplasm of neurons and most nuclei with either chromatin margination or hyperchromatic darkly stained appearance (Figure 8(b)). RB+quercetin cotreatment in group III, on the other hand, significantly ameliorated the previously observed destructive alterations associated with RB, and the cerebral cortex appeared to have been preserved, apart from a few neurons with faint cytoplasm or scattered vacuolation (Figure 8(c)). The cerebral cortex of rats given RB and allowed to recover for two weeks in group IV showed faint cytoplasm and chromatin margination in the utmost nuclei of neurons, as well as broad areas of neuropil disintegration in between (Figure 8(d)).

When compared with the control group or the QR-treated group, the percentage of preserved neurons in group II was significantly lower (*P* < 0.01) by 53% and 51%, correspondingly. A statistically nonsignificant difference (*P* > 0.05) was observed when comparing group III treated with quercetin to the control group, indicating that the percentage of intact neurons in this group is higher. Furthermore, group III was one fold and 43% higher than groups II and IV, respectively, while group IV (RB-recovery) showed a statistically substantial upregulation (*P* < 0.01) in the percentage of intact neurons when compared to the RB-treated group by 41%, but it still revealed a significant decrease (*P* < 0.01) in the percentage of intact neurons when compared to either the control by 33% or QR-supplemented by 30% (Figure 8(e)).

### 3.9. Immunohistochemical Results of Neuron-Specific Enolase

**Figure 9:**
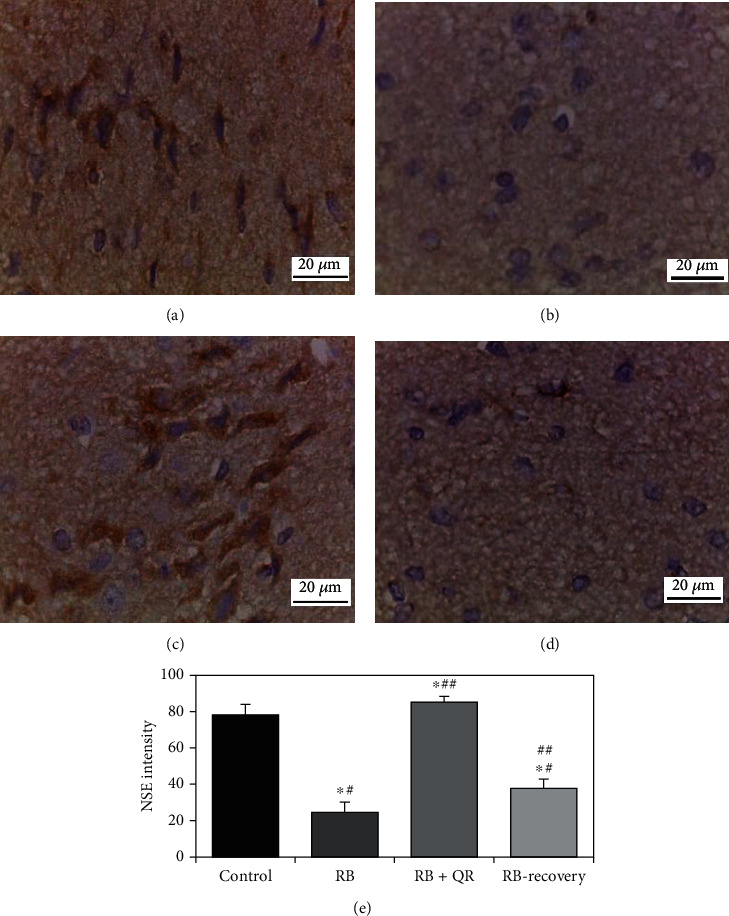
Impact of quercetin on NSE immunohistochemical expression (scale bar: 20 *μ*m): (a) the control group, (b) the RB group, (c) the RB+QR cotreated group, (d) the RB-recovery group, and (e) the intensity of NSE immunoreactive neurons in the cerebral cortical tissue of all groups. ^∗^*P* < 0.01 vs. the control group; ^#^*P* < 0.01 vs. the RB+QR group; ^##^*P* < 0.01 vs. the RB group.

The NSE immunoreaction was detected positively in the neuronal cells from the control group (Figure 9(a)). In the cerebral cortex of either group II (RB-treated) (Figure 9(b)) or group IV (RB-recovery) (Figure 9(d)), the NSE immunoreaction displayed reduction. In group III (RB+QR concomitant administration), however, there was an increase in the NSE immunoreactivity expression (Figure 9(c)).

The percentage of NSE immunopositive neuronal cells in the RB+QR concomitantly administered rats increased statistically (*P* < 0.01) as compared to either group II (RB-treated) by 2 folds or group IV (RB-recovery) by one fold. At the same time, when compared to the control group, both groups II and IV had a statistically considerable decrease (*P* < 0.01) in the percentage of NSE immunoreaction by 68% and 52%, respectively. Moreover, there was a significant increase when comparing both QR cotreated with the control group by 9% (*P* <0.05) and the recovery group with the RB-treated one by 54% (*P* <0.01) (Figure 9(e)).

### 3.10. Correlation Results between the Different Studied Parameters

There was significant negative correlation between BDNF and IL-1*β* (*r* = −0.733, *P* < 0.001), Nrf2 and TBARS (*r* = −0.706, *P* < 0.001), Nrf2 and IL-1*β* (*r* = −0.685, *P* < 0.001), Nrf2 and HSP70 (*r* = −0.698, *P* < 0.001), Nrf2 and 8-OHdG (*r* = −0.661, *P* < 0.001), TAC and HSP70 (*r* = −0.657, *P* < 0.001), TAC and 8-OHdG (*r* = −0.640, *P* < 0.001), and TBARS and BDNF (*r* = −0.821, *P* < 0.001), while significant positive correlation was noticed between TBARS and IL-1*β* (*r* = 0.828, *P* < 0.001), Nrf2 and TAC (*r* = 0.832, *P* < 0.001), Nrf2 and HO-1 (*r* = 0.801, *P* < 0.001), Nrf2 and BDNF (*r* = 0.622, *P* < 0.001), and TBARS with both HSP70 (*r* = 0.8, *P* < 0.001), and 8-OHdG (*r* = 0.722, *P* < 0.001).

## 4. Discussion

In the current study, the concentration of TBARS as a measure of oxidative stress was analyzed, and it was discovered that group II treated with RB-Ed and group IV left to recover after RB-Ed treatment for 4 weeks had a substantial rise. However, in both groups II and IV, the content of TAC, an antioxidant indicator, was significantly lower. The brain is especially sensitive to oxidative harm due to its high polyunsaturated fat content and aerophilic metabolism. Many components of RB-Ed can cross the blood-brain barrier (BBB) due to their high affinity for lipid solubility [28], causing fatty acid oxidation and inflammatory reactions, as measured by an increase in oxidative items and proinflammatory cytokines [29], as confirmed in the current study by a strong positive correlation between TBARS and IL-1*β*.

When compared to Red Bull-supplied groups II and IV, there was an improvement in the redox status of group III cotreated with quercetin, as evidenced by a considerable downregulation in the lipid peroxidation marker TBARS and an elevation in the antioxidant marker TAC. Sharma et al. [30] published similar findings, claiming that QR reduces the oxidative stress in rat tissues. Chen et al. [31] emphasized the antioxidant impact of QR by accentuating its involvement in decreasing lipopolysaccharide-induced nitric oxide generation in a mouse neuroglial cell line. Furthermore, Swapnila et al. [32] revealed that QR is a more effective antioxidant than other antioxidative nutrients like vitamin E, beta-carotene, and vitamin C on a molar basis. The positive benefits of quercetin were attributed by Nabavi et al. [33] for its direct scavenging of free radicals and indirect stimulation of endogenic antioxidant production. QR's free radical scavenging activity is based on its basic design, according to Federico et al. [34]. QR also reduced protein carbonyls, a global indicator of oxidative injury to proteins' amino acids, according to Nageshwar et al. [35].

In this study, RB intake resulted in lower levels of the mRNA and protein expression of Nrf2 and HO-1 genes in group II treated with RB-Ed as compared with the control group. However, in group III, when QR was given in combination with RB, the quantities of Nrf2 and HO-1 genetic expression in the cerebral cortical tissues were considerably higher than in the other groups. Furthermore, compared to the normal group or the RB+QR concurrently treated rats, group IV, which was administered RB for 4 weeks and then rested 2 weeks to recuperate, had significantly lower levels of Nrf2 and HO-1. Na and Surh [36] stated that translocation of activated Nrf2 to the nucleus enhanced the transcription of phase II enzyme genes including heme oxygenase 1. Rangasamy et al. [37] pointed out that considerable exposure to chemical risk linked to oxidative stress causes Nrf2 or HO-1 downregulation in mice, like the findings of this study. Furthermore, according to Soares and Bach [38], HO-1 and its derivatives protect against a variety of toxins.

According to Block et al. [39], Nrf2 is the primary controller of the oxidative stress-induced inflammatory response in the cerebral cortex. This finding is consistent with the results of the current study, which found a strong negative connection between Nrf2 and both TBARS and IL-1*β*. The current study further emphasized the strong positive association between Nrf2 and TAC. According to Son et al. [40], an increase in Nrf2 levels enhances the expression of antioxidant and detoxifying enzymes in brain cells, acting as a protective mechanism. Additionally, in accordance with Sandberg et al. [41], a functioning Nrf2 system can be a crucial regulator of oxidative stress-induced inflammation in the brain. As indicated by the current study's finding of a considerable positive association between Nrf2 and HO-1, Ahmed et al. [14] characterized Nrf2 as a crucial transcript element that controls oxidative stress and inflammation by stimulating several phase II antioxidant enzymes, including heme oxygenase-1 (HO-1).

When compared to both normal and RB+QR-cotreated rats, Red Bull caused a significant decrease in brain-derived neurotrophic factor (BDNF) concentration and a significant increase in the proinflammatory cytokine IL-1*β* level in the cerebral cortex of group II, and these findings were replicated in group IV after two weeks of recovery. Shi et al. [42] validated these findings, reporting that BDNF was downregulated in both acute and chronic stress.

Consistent with Lapchak et al. [43], the proinflammatory cytokines can diminish the production of BDNF in mouse cerebral cortical tissues, showing their impact on neuronal homeostasis, as signified by the current study's strong negative connection between BDNF and IL-1*β*. Furthermore, as demonstrated in the present work's Red Bull-treated group II and RB-recovery group IV, an increase in proinflammatory cytokines might cause neuronal cell degeneration, in line with Diaz et al. [29].

Zuccato et al. [44] pointed out that BDNF can manipulate and enhance neurogenesis in the central and peripheral nervous systems. Furthermore, Song et al. [45] explained how BDNF is important for synaptic malleability, neuron survival, differentiation, and renewal. Moreover, Lu et al. [46] defined BDNF as a protein that is important for studying advanced reasoning, memory, and the beginning of thought.

In group III of the current investigation, concurrent quercetin treatment resulted in a significant decrease in IL-1*β* and an increase in BDNF. According to Sharma et al. [47], QR inhibits the production of cytokines. QR has also been investigated as a potential safe alternative to anti-inflammatory and antioxidant drugs for a variety of ailments [35]. According to Costa et al. [48], the architectural and molecular procedures for QR's anti-inflammatory effects remain unknown, although a probable path could be linked to paraoxonase 2 (PON2) stimulation, which has anti-inflammatory and antioxidant activity.

In this study, the Red Bull-administered group II had histomorphological degenerative results like cytoplasmic vacuolation, darkly reduced nuclei, and chromatin lysis, as well as a reduction in granular appearance resulting in light cytoplasm. Furthermore, the two-week recuperation period had no effect on the results in group IV. The current study's histomorphological changes were based on the findings of Bawazir and Almehmadi [49], who reported atrophy of neurons and nuclei after daily Red Bull energy drink administration. Steward [50] observed that, like the present study's findings, chromatin breakdown halted the production of neuronal proteins. Gepdiremen et al. [28] previously linked the negative effects of RB-Ed to one of its most essential constituents, caffeine, which can pass across the blood-brain barrier because of its strong affinity for lipid solubility. Yang et al. [51] further linked Nrf2's cellular protective role against oxidative and toxicant-induced damage to its translocation into the nucleus and binding to antioxidant response elements (ARE), a transactivating set as cytoprotective enzymes.

In the current study, the histomorphological architecture of the cerebral cortex in the RB+QR-cotreated group was significantly improved when compared with the other groups. According to Yuhan et al. [52], quercetin, a polyphenolic flavonoid, has received interest due to its antioxidant quality. Quercetin also protects against toxicant-induced histoarchitectural alterations, in accordance with Jiang et al. [53].

Furthermore, the observed histomorphological improvement was confirmed in the current study not only by an increase in the percentage of NSE immunoreactive neurons with coadministration of QR in group III, which showed a statistically significant upregulation when compared to the RB-recovery group, but also by an increase in the percentage of preserved neurons in toluidine blue-stained sections. In comparison to the control and RB+QR-treated groups, both group II (RB-treated) and group IV (RB-recovery) had lower percentage counts of immunopositive NSE neuronal cells in the cerebral cortex.

Compliant with Asa et al. [16], NSE is an acid-soluble enzyme that catalyzes glucose and is employed as a potent marker of metabolic function in cells *in vivo* and *in vitro*. QR administration increased NSE immunoreactivity, along with Nogami et al. [54], which was like the findings of this study. The authors stated that NSE immunopositivity is preserved in less degraded neurons, and NSE cerebral cortex immunoreaction is beneficial in detecting many situations of brain damage. As a result, the existence of more NSE immunoreactive neuronal cells in group III treated with quercetin of the current investigation implies that the cerebral cortex is becoming more active. Furthermore, Zhou et al. [55] revealed a relationship between changes in NSE activity levels and neuronal histomorphological abnormalities.

HSP70 and 8-OHdG concentrations in the cerebral cortex of rats increased in groups II and IV compared to the other groups. Furthermore, quercetin treatment concurrently with RB in group III significantly reduced the rise in HSP70 and 8-OHdG levels when compared to RB-treated rats in group II or the RB-recovery group. Furthermore, the current study found that RB administration resulted in an increase in the creation of TBARS oxidative elements as well as a deficiency in TAC antioxidant markers, which could lead to histomorphological detrimental molecular consequences such as nucleotide bond concentration. Furthermore, the present study discovered a strong positive correlation between TBARS and DNA degradation markers, HSP70 and 8-OHdG. According to Bloomer et al. [56], a decline in the antioxidative system caused injury to neuronal cell items such as proteins, lipids, and DNA arrangements, leading to cell death. As mentioned by Ayer and Zhang [57], reactive oxygen species increase lipid peroxidation and DNA injury, resulting in further neuron cellular degeneration. The presence of a substantial negative connection between TBARS and BDNF protein was demonstrated in the present work. Ming et al. [58] added that guanine is particularly susceptible to chemical reactions because of its low oxidative-reducing ability; 8-hydroxy-2-deoxyguanosine is the most common type of deoxyribonucleic acid product, and its level is used as an assistant marker of oxidative deoxyribonucleic acid defacement.

## 5. Conclusion

In conclusion, when taken in combination with RB-Ed, quercetin appears to operate as a neuroprotective agent against RB-induced oxidative damage by boosting the TAC capacity levels and protecting against TBARS via upregulation of Nrf2 and HO-1. Moreover, QR significantly reduces Red Bull-induced cellular degeneration of the cerebral cortex which is confirmed by the reduction in the damaged neurons and an increase in NSE immunoreacted neurons, suggesting quercetin's neuroprotective function against RB-Ed damage. QR ameliorates the reduction of BDNF and acts as an anti-inflammatory agent by reducing the proinflammatory marker, IL-1*β* and DNA damage markers, and HSP70 and 8-hydroxydeoxyguanosine. As a result, it is critical to include quercetin in the diet to prevent neurodegenerative changes caused by long-term RB-Ed consumption, as the time spent recovering after receiving RB-Ed had no effect on reducing the deleterious effects.

## Data Availability

The datasets used during the current study are available from the corresponding author on reasonable request.
